# Impact of Rechallenge with Imatinib in Patients with Advanced Gastrointestinal Stromal Tumor after Failure of Imatinib and Sunitinib

**DOI:** 10.1155/2014/342986

**Published:** 2014-01-22

**Authors:** Akira Sawaki, Tatsuo Kanda, Yoshito Komatsu, Toshirou Nishida

**Affiliations:** ^1^Department of Gastroenterology, Aichi Cancer Center Hospital, 1-1 Kanokoden, Chikusa-ku, Nagoya-shi, Aichi 464-8681, Japan; ^2^Digestive and General Surgery 2 Division, Niigata University Hospital, 754 Ichibancho, Asahimachidori, Chuo-ku, Niigata-shi, Niigata 951-8510, Japan; ^3^Department of Cancer Chemotherapy, Hokkaido University Hospital, Cancer Center, Kita 14-jo Nishi 5-chome, Kita-ku, Sapporo-shi, Hokkaido 060-8648, Japan; ^4^Department of Surgery, Osaka University, School of Medicine, 2-2 Yamadaoka, Suita-shi, Osaka 565-0871, Japan

## Abstract

*Purpose*. This retrospective, nonrandomized study investigated the effect of imatinib rechallenge plus best supportive care (BSC) on overall survival after imatinib and sunitinib treatment for patients with locally advanced or metastatic gastrointestinal stromal tumor (GIST). *Methods*. Twenty-six patients who had previously been exposed to both imatinib and sunitinib were enrolled in this study. The treatment regimen was BSC with or without imatinib, based on the patient's choice after discussion with his or her physician. The primary endpoint was overall survival, and secondary endpoints were time to treatment failure, clinical response rate assessed by Choi criteria, and safety. *Results*. Fourteen patients were treated with imatinib plus BSC and 12 received BSC alone. Median overall survival was greatly improved for the imatinib group, although differences were not significant (22 months for imatinib plus BSC versus 4 months for BSC; *P* = 0.058). Three patients (21%) had a clinical response in the imatinib group, and one had a clinical response in the BSC alone group. Imatinib was well tolerated. *Conclusions*. Rechallenge with imatinib may be associated with improvement in overall survival without deteriorating performance status in patients who failed imatinib and sunitinib. A prospective study should be considered to confirm the efficacy of rechallenge with imatinib.

## 1. Introduction

Gastrointestinal stromal tumor (GIST) is the most common type of nonepithelial tumor occurring in the gastrointestinal (GI) tract, including the peritoneum [1]. GISTs cannot always be distinguished from myogenic or neurogenic neoplasms by routine histological methods; as such, immunohistochemistry is often needed to distinguish GISTs from other spindle cell tumors. GISTs are generally considered to be KIT (CD117) positive tumors [2]. The tumor probably arises from KIT or platelet-derived growth factor receptor A (PDGFRA) gene mutations in precursor cells that normally give rise to the interstitial cells of Cajal [3–5]. Some GISTs are clinically malignant and metastasize predominantly to the liver and peritoneum [1, 6, 7]. About 85–90% of GISTs are associated with gain-of-function KIT gene mutations that lead to constitutive activation of KIT kinase activity [3]. A much smaller proportion of GISTs are associated with analogous gain-of-function mutations in PDGFRA, the gene-encoding platelet-derived growth factor receptor *α* (PDGFR*α*); less than 10% contain no identified receptor tyrosine kinase mutations [8, 9]. Activating mutations of KIT or PDGFRA have been identified in the development and maintenance of the malignant phenotype in most cases of GISTs [3, 8, 9].

Imatinib mesylate, a selective inhibitor of the kinase activities of KIT and PDGFRA, has substantially improved clinical outcomes for patients with advanced disease [10–12]. However, in a pivotal study of imatinib in advanced GIST, 5% of patients were intolerant of imatinib and another 14% developed early resistance [10]. Secondary or acquired resistance develops after a median of about 2 years [12]. Sunitinib malate is an oral multitargeted receptor tyrosine kinase inhibitor that has shown antiangiogenic and antitumor activities in several *in vitro* and *in vivo* tumor models [13–18]. A phase 3 study [19] demonstrated significant clinical benefit, including disease control and superior survival, with sunitinib compared with placebo in patients with advanced GIST after failure of imatinib. The primary resistance rate of sunitinib was more than 50%, and progression-free survival of sunitinib was approximately 7 months [19]. Regorafenib is a multikinase inhibitor that blocks targets and receptors including VEGFR1–3, TIE2, PDGFR, FGFR, KIT, and RET, which are associated with angiogenesis and tumor progression. A phase 3 study [20] found that patients who received regorafenib had a period of progression-free survival of 4.8 months compared with 0.9 months in patients who received a placebo (hazard ratio (HR) = 0.27; 95% CI, 0.19–0.39;  *P* < 0.0001). Regorafenib is available in limited countries for patients with GIST that had become resistant to imatinib and sunitinib.

Effective alternative treatments after failure of imatinib and sunitinib therapy are an important unmet medical need. If there is no chance of receiving a new drug being studied in a clinical trial, some patients choose to receive imatinib with best supportive care (BSC). In the European Society for Medical Oncology (ESMO) guidelines, Casali et al. state that patients who have already progressed on imatinib may occasionally benefit when rechallenged with the same drug [21]. To date, there are no reports on imatinib plus BSC after failure with both imatinib and sunitinib. Thus we performed a retrospective study to compare the clinical significance of imatinib plus BSC with BSC alone in patients with advanced GIST after failure of imatinib and sunitinib.

## 2. Patients and Methods

### 2.1. Patients

This retrospective study included patients who were registered in a postmarketing study of sunitinib for GIST at Osaka University Hospital, Niigata University Hospital, Hokkaido University Hospital, and Aichi Cancer Center Hospital between June 2008 and March 2009. Patients included in the study had to have immunohistologically or molecularly proven, locally advanced, or metastatic GIST that was not curable with surgery or any other therapy; had previously received at least imatinib or other chemotherapy regimens and were refractory to their latest chemotherapy regimen; had Eastern Cooperative Oncology Group (ECOG) Performance Status (PS) of 0-1 (those with a PS of 2 were also eligible if the investigator believed that poor PS was not predominantly due to comorbidity); and had adequate bone marrow, renal, and liver functions.

Exclusion criteria were as follows: less than 1 week since completion of previous radiotherapy or persistence of any radiotherapy-related toxic effects; unresolved chronic toxic effects from previous anticancer therapy; severe or uncontrolled systemic disease including cardiac disease or clinically active interstitial lung disease; and concomitant use of phenytoin, carbamazepine, rifampicin, barbiturates, or St. John's wort.

Of the recruited patients, those who were still being treated with sunitinib in March 2009 and participated in another clinical trial after the end of sunitinib treatment were excluded. Our retrospective study was approved by the Ethics and Scientific Committee of Aichi Cancer Center Hospital, which did not require written informed consent because this study was an epidemiological investigation.

### 2.2. Study Design and Treatment

Patients were given imatinib (400 mg/day) plus BSC or BSC only; treatment was selected by the patient after discussion with a physician (i.e., there was no randomization). All patients received BSC according to the local practice of the individual institutions. Patients continued imatinib until unacceptable toxic effects occurred or the patient was no longer deriving clinical benefit.

The primary endpoint was overall survival, and secondary endpoints were time to treatment failure, clinical response rate assessed by Choi criteria, and safety. Overall survival was assessed from the date of sunitinib discontinuation to the date of patient death; patients who were alive at data cutoff were censored in the overall survival analysis. Time to treatment failure was calculated as the time from the date of sunitinib discontinuation to the date at which the patient discontinued therapy due to (a) unacceptable toxic effects; (b) no further clinical benefit (assessed by an investigator); (c) the patient's choice; or (d) death from any cause. Tumor progression (as defined by Response Evaluation Criteria In Solid Tumors (RECIST)) was not necessarily classified as treatment failure; patients could continue to receive treatment as long as they continued to derive clinical benefit. Patients who had not failed treatment at data cutoff were censored for time to treatment failure at the time of their last visit. Tumors were assessed at baseline; the specific imaging modality was at the discretion of the investigator. Subsequent imaging was regularly undertaken, but duration of radiological monitoring was decided by investigators. Clinical response was evaluated according to Choi criteria on computed tomography (CT) at 2 months after treatment [22, 23]: a decrease in tumor size of ≥10% or a decrease in tumor density of ≥15% was considered a clinical response. Those who were not monitored by radiological study were excluded from the response evaluation. Adverse events were monitored and graded by the Common Terminology Criteria for Adverse Events (version 3.0) of the National Cancer Institute. Routine laboratory monitoring (including biochemistry, hematology, and urine analysis) was also performed.

### 2.3. Statistical Analysis

Time to treatment failure and overall survival were calculated using the Kaplan-Meier method. Overall survival between treatment groups was compared using the stratified log-rank test. All analyses were performed using SPSS version 12 (SPSS, Chicago, IL, USA) statistical software. Statistical results were considered significant at *P* < 0.05. All reported *P* values are two-sided.

## 3. Results 

A total of 47 patients were identified from four Japanese institutions. Eight patients were still being treated with sunitinib therapy, and 13 patients were participating in another clinical trial. Consequently, 26 patients treated with imatinib plus BSC or BSC alone were included for the study. Baseline characteristics were not balanced between the two treatment groups (Table 1). The imatinib plus BSC group was younger than the BSC group; in addition, more patients with poor PS (≥2) were in the BSC group, whereas there were no statistically significant differences in both factors. Patients whose PS worsened from presunitinib to postsunitinib were significantly more likely to choose BSC. Duration of their first imatinib treatment in the imatinib plus BSC group appeared to be longer than that in the BSC group, although differences were not significant. In each group, half or more of patients showed a tumor response to first-line imatinib treatment; all but one stopped therapy due to resistance. The treatment duration of second-line sunitinib was 7.5 and 6.0 months in the imatinib plus BSC and BSC groups, respectively.

**Figure 1:**
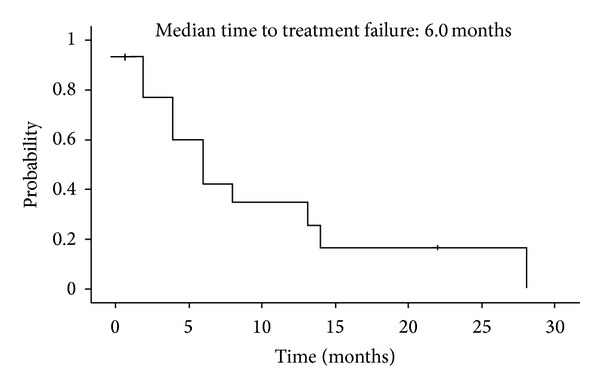
Time to treatment failure in all enrolled patients. Median time to treatment failure was 6.0 months.

**Figure 2:**
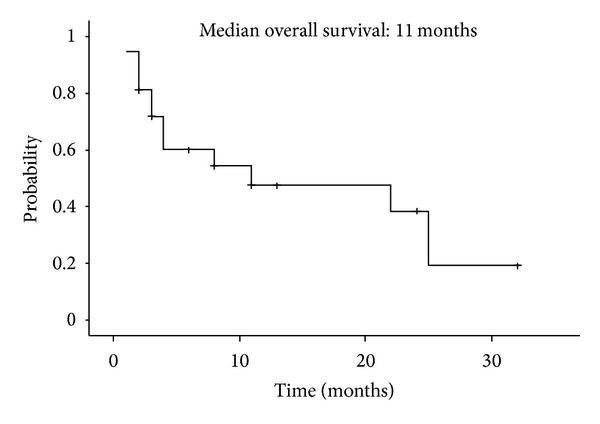
Overall survival in all enrolled patients. Median overall survival time was 11 months.

**Figure 3:**
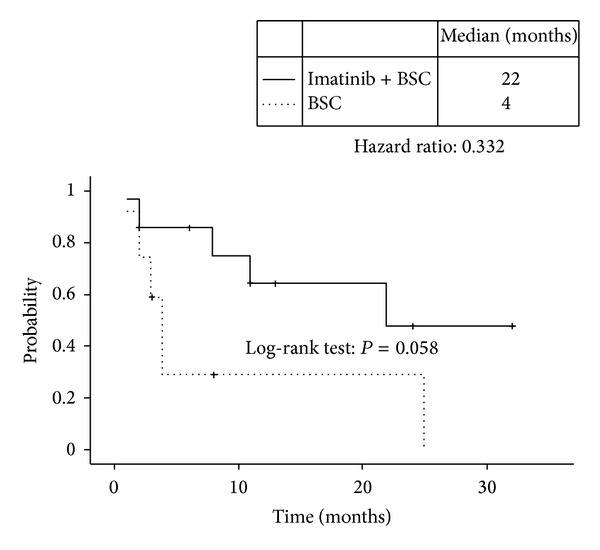
Overall survival curves in the imatinib plus BSC group (solid line) and the BSC alone group (dotted line). Median overall survival times were 22 months and 4 months, respectively. The hazard ratio for imatinib plus BSC was 0.332 (*P* = 0.058, log-rank test). BSC, best supportive care.

At data cutoff (March 31, 2009), median followup was 7.2 months and median time to treatment failure for all enrolled patients was 6.0 months (Figure 1). The overall survival from the date of sunitinib discontinuation was 11 months (Figure 2). Median overall survival in the imatinib plus BSC and BSC groups, respectively, was 22 months and 4.0 months (Figure 3). The survival curves apparently separated at 18 months for median survival time (hazard ratio (HR) = 0.332; 95% confidence interval (CI), 0.103–1.070), but the differences between the two groups did not reach statistical significance in the log-rank test for overall survival (*P* = 0.058).

In total, tumor response was evaluable at baseline in 14 patients (Table 2). The clinical response rate was higher in the imatinib plus BSC group (3 of 14; 21%) than in the BSC group (1 of 12; 8%), but most patients in both groups were not evaluable.

All 26 patients were evaluable for tolerability (Table 3). All patients in the imatinib plus BSC group and all but two patients in the BSC group experienced at least one adverse event. However, these adverse events were grade 1 or 2 in severity; no grade 3 or 4 adverse events were observed. The most common adverse events were periorbital edema (Table 3). Anemia and liver dysfunction appeared more in the BSC than in the imatinib plus BSC group. Dose interruptions due to adverse events were necessary in three (21%) patients. Leukopenia and neutropenia were generally asymptomatic and mild in severity.

## 4. Discussion

Median overall survival times in the imatinib plus BSC and BSC groups, respectively, were 22 and 4 months (HR: 0.332); survival curves (Figure 3) apparently separated after 2 months. This result supports the clinical benefits of rechallenging patients with imatinib. Imatinib rechallenge was well tolerated. Although patients who were rechallenged with imatinib experienced at least one adverse event, most adverse events were mild, and the frequency of adverse events was low compared to that occurred during initial treatment with imatinib. The ESMO guidelines recommend that rechallenge or continuation of treatment with antityrosine kinase agent may be an option on individual basis [21] because there is anecdotal evidence that patients who have already progressed on imatinib may occasionally benefit when rechallenged with the same drug. On the other hand, the National Comprehensive Cancer Network (NCCN) guidelines [24] strongly recommend participating in a clinical trial or receiving BSC if a patient is no longer obtaining clinical benefit from imatinib and sunitinib. The NCCN guidelines do not discuss rechallenging patients with 400 mg of imatinib after failure to respond to imatinib and sunitinib, most likely because studies on rechallenging were scarce. Our results support the ESMO recommendations: after treatment failure with imatinib and sunitinib, some patients might experience longer survival with less toxicity if rechallenged with imatinib.

Our results are also consistent with data from recent studies that evaluated the effect of imatinib interruption and rechallenge in patients with advanced GIST. In clinical practice, many patients choose to interrupt therapy, with or without their physicians' knowledge, due to recurrent toxicities, concomitant comorbidities, or the need for a break from therapy. However, studies have shown that imatinib interruption in advanced GIST patients could result in rapid tumor progression in the majority of the patients [25, 26]. Imatinib rechallenge reestablished tumor control in most patients [25, 26], but the tumor response was not as good as that achieved prior to treatment interruption [27]. In our study, response after imatinib rechallenge also appeared to be inferior to that observed during initial imatinib treatment; however, this conclusion is limited by the fact that tumor response was only evaluable in 14 patients.

The mechanism for response to secondary treatment with imatinib is heterogeneity [28] of metastatic lesions. Proliferation of GIST cells is predominantly driven by KIT or PDGFRA signaling, and 44–67% of resistance to imatinib [29–32] was shown to be due to a secondary mutation. This additional mutation causes the loss of affinity to imatinib, subsequently leading to resistance to therapy. However, oftentimes not the whole tumor is composed of resistance clones. Heinrich et al. [8] reported the presence of sensitive mutations in exon 11 in the c-KIT gene or exon 12 in *PDGFRA* genes and resistance clones in exon 11 plus exon 13 or exon 14 in the c-KIT gene. Rechallenge with imatinib may demonstrate prolongation of time to treatment failure and overall survival if some lesions remain sensitive. Our favorable results may be caused by this heterogeneity of mutation status after resistance to imatinib and sunitinib. Even in lesions with double mutations that show extreme imatinib resistance, imatinib may weakly inhibit the downstream signaling of KIT or PDGFRA and slows tumor progression [29]. Imatinib might also have a clinical benefit in patients with primary resistance to imatinib.

This study has several limitations: it was retrospective and nonrandomized and included a small number of centers and patients. Rechallenge with imatinib was chosen based on the discussion between the patient and doctor. Medical doctors were more likely to recommend imatinib rechallenge for patients with a better general status and patients who responded better to initial imatinib therapy. For example, patients who were rechallenged with imatinib tended to be younger and have better PS scores (Table 1). The treatment period of initial imatinib therapy in the rechallenge group (41 months) was about twice as long as that in BSC group (19.5 months). Despite these limitations, our results suggest that, for patients who fail therapy with imatinib and sunitinib, if no trial is available, rechallenge with imatinib might be a feasible choice. Patients who were treated with the initial imatinib therapy for >40 months and who have a better PS may be good candidates for imatinib rechallenge.

In conclusion, rechallenge with imatinib might have benefit for some patients with better PS and a long exposure to initial imatinib therapy. If new agents are not available, rechallenge with imatinib should be considered. However, a prospective study is warranted to confirm the efficacy of rechallenge with imatinib.

## Figures and Tables

**Table 1:** Baseline characteristics.

Characteristic	Imatinib + BSC (*n* = 14)	BSC alone (*n* = 12)	*P* value^†^
Age (years)			
Median (range)	54.5 (48–7)	62.5 (41–73)	—
Sex			
Male	8	8	0.701
Female	6	4	
ECOG PS			
1	8	4	0.220
2	6	5	
3	0	1	
4	0	2	
Change in PS*			
Better	1	0	0.003
Worse	1	8	
No change	12	4	
Primary site of cancer			
Stomach	5	3	0.913
Small intestine	7	7	
Rectum	1	1	
Other	1	1	
Metastatic sites			
Liver	5	8	0.728
Peritoneum	9	9	
Other	3	2	
Duration of initial treatment with imatinib, months			
Median (range)	41 (20–79)	19.5 (1–49)	—
Best response to initial treatment with imatinib			
CR	3	4	0.351
PR	3	2	
SD	1	2	
PD	3	4	
NE	4	0	
Reason for cessation of initial imatinib treatment			
Resistance	14	11	0.462
Intolerance	0	1	
Duration of sunitinib therapy, months			
Median (range)	7.5 (3–17)	6 (1–34)	—

BSC: best supportive care; ECOG PS: Eastern Cooperative Oncology Group Performance Status; CR: complete response; PR: partial response; SD: stable disease; PD: progressive disease; NE: not evaluable.

*Compared with presunitinib status.

^†^Fisher's exact test.

**Table 2:** Clinical response.

Response	Imatinib + BSC (*n* = 14)	BSC alone (*n* = 12)
Responder*	3	1
Nonresponder^†^	6	4
Not evaluable	5	7

BSC: best supportive care.

*Observed clinical response by Choi criteria.

^†^No observed clinical response by Choi criteria.

**Table 3:** Adverse events.

Toxicities	Grade 1		Grade 2		Grade 3/4	
	IM + BSC	BSC	IM + BSC	BSC	IM + BSC	BSC
Anemia	2	3	0	2	0	0
Leukopenia	2	1	4	0	0	0
Neutropenia	3	0	0	0	0	0
Thrombocytopenia	2	1	1	0	0	0
Liver dysfunction	1	3	1	1	0	0
Skin rash	1	0	0	0	0	0
Edema	12	4	1	0	0	0
Nausea	3	2	1	0	0	0
Anorexia	3	2	0	0	0	0
Diarrhea	3	2	0	0	0	0

IM: imatinib; BSC: best supportive care.
